# Medical ID use by international patients with Aspirin-Exacerbated Respiratory Disease

**DOI:** 10.1186/s13223-023-00766-7

**Published:** 2023-03-13

**Authors:** Mohammed Alqabasani, Andrea Lasso, Shaun Kilty

**Affiliations:** 1 Department of Otolaryngology-Head and Neck Surgery, University of Ottawa - 501 Smyth Rd, Ottawa, ON Canada; 2 Ottawa Hospital Research Institute, 1053 Carling Ave, Ottawa, Canada; 3 Department of Otolaryngology-Head and Neck Surgery, University of Ottawa, The Ottawa Hospital. Ottawa Hospital Research Institute, 1053 Carling Ave, 259-737 Parkdale Ave, Ottawa, ON Canada

**Keywords:** Emergency medical tag, Aspirin induced asthma, NSAID-induced asthma, Chronic rhinosinusitis with polyps

## Abstract

**Background:**

Patients widely use medical identification (ID) to indicate their food and drug allergies, and chronic medical conditions. One chronic condition for which patients are recommended to use a form of medical ID is Aspirin-Exacerbated Respiratory Disease (AERD), a disease characterized by the presence of asthma, chronic rhinosinusitis with nasal polyps and sensitivity to aspirin and other COX-1 inhibitors, including nonsteroidal anti-inflammatory drugs (NSAIDs). The uptake of medical ID use in AERD is unknown and has not been widely studied in this population.

**Methods:**

We conducted a cross-sectional survey study to measure the perception of the need to use a medical ID and its use by patients with AERD internationally.

**Results:**

245 members of an online AERD support group completed an online survey. The majority (80%, n = 198) of the participants did not use any form of medical ID. The participants reported that the lack of knowledge and awareness about the importance of using a medical ID was the most common reason for not using it.

**Conclusion:**

This international survey found that the majority of the AERD patient respondents did not use a medical ID. The most common reasons for nonuse were not knowing that it is recommended for their condition and that the patients did not consider it necessary. The results highlight the need for further patient and health care provider education.

**Supplementary Information:**

The online version contains supplementary material available at 10.1186/s13223-023-00766-7.

## Background

Patients widely use medical identification (ID) to indicate their food and medication allergies as well as any chronic medical conditions. One of the chronic conditions for which patients are recommended to use a form of medical ID is Aspirin-Exacerbated Respiratory Disease (AERD) [1]which is diagnosed by the presence of asthma, chronic rhinosinusitis with nasal polyps and a sensitivity to aspirin and other cyclooxygenase-1 (COX-1) inhibitors, including nonsteroidal anti-inflammatory drugs (NSAIDs). AERD usually presents during the third and fourth decades of life and is more common in females [1]. Almost half of those with AERD have severe asthma, and exposure to NSAIDs can lead to a serious acute asthma exacerbation. [2, 3]

AERD patients who have not been desensitized must avoid aspirin and NSAIDs. However, total avoidance has been shown to be difficult to achieve. In a survey of AERD patients, 24% reported that they had accidentally ingested an NSAID after they were diagnosed with AERD and were made aware that they should avoid these medications [4]. In twenty five percent of the cases, the medication had been prescribed, with the highest incidence of NSAID prescription by emergency room physicians and other non-ENT surgeons. In an emergency, when patients cannot provide medical history, they are at risk of unintentional exposure to these medications. One example is the use of aspirin as a pre-hospital treatment for chest pain of suspected cardiac origin [5, 6]. Accidental NSAID exposure in emergency circumstances may be avoided by using a medical ID, considering that 97% of ambulance staff and 71% of emergency room personnel check routinely for a medical alert ID [7].

Recently, our team evaluated the prevalence of use of medical ID among patients with AERD at our institution [8]. We surveyed 21 patients with AERD and found that only 19% of patients wore a medical ID. Sixty percent of those who did not wear an ID cited not knowing they should wear one as the primary reason for nonuse. Although this survey was small, to our knowledge, this was the first attempt to determine the extent of use of medical ID in this at risk population. With these results, we sought to evaluate medical ID use in a larger population of AERD patients.

The goal of the present study was to determine the proportion of an international group of patients previously diagnosed with AERD who are currently using a medical ID and to explore patients’ attitudes toward the use of medical ID.

## Methods

### Ethical considerations

Ethical approval for this study was obtained from the Ottawa Health Sciences Network Research Ethics Board (REB# 20200627-01H). This cross-sectional study used an anonymous online survey created on LimeSurvey [9] hosted at the Ottawa Hospital. The questionnaire was designed to measure the perception of the need to use medical ID and the actual behaviour, use of a medical ID by a patient diagnosed with AERD.

The questionnaire included two parts. Forced choice questions were used for most of the survey. The first part of the questionnaire collected participant demographic data, the perception of medical ID need, its use, the type of ID used and the duration of use. The second part of the questionnaire was for the patients who reported not using a medical ID. The reasons for not using a medical ID were recorded using both forced response and open response options. A link to the survey was posted on the Samter’s Society: AERD Samter’s Triad Support Group on Facebook with the administrator's approval. The survey was open between November 17, 2021, and March 2, 2022, and 2 reminders were posted on the group’s site during this time. The goals of the study were explained on the first screen, and completion of the survey implied consent to participate in the study. We present a descriptive analysis of the participants’ characteristics. Chi-square tests were used to test associations between demographic characteristics and the use of a medical ID. Analysis was done using STATA software (Version 12. College Station, TX: StataCorp LP).

## Results

A total of 245 members of the support group completed the survey. The average age at the time of the survey was 51 years (SD = 12.51); 215 participants (87.76%) were female. Most respondents were located in North America (77.96%) and Europe (14%). Sixty Five percent of respondents had a bachelor’s degree or higher, and 125 (51%) reported a household income of > 80,000 USD per year (see Table 1). Sixty-one (25%) of respondents reported being aware that for people with AERD, it is recommended to use a medical ID; however, only 47 (19%) of the patients were using a medical ID at the time of the survey. Of these, 28 (59.5%) reported using a bracelet, 15 (25.5%) reported using smartphone technology (see Table 2). Twenty-nine (61.7%) of those who were wearing an ID had been wearing it for 1–5 years, while 11 (23.4%) had been wearing the ID for 5–10 years; 7 respondents (14.8%) had been wearing the ID for less than a year. The majority of the participants (n = 198, 81%) were not using any form of medical ID at the time of the survey. The most common reason for not using medical ID was a lack of knowledge regarding the importance of its use. Other patient reasons are listed in Table 3. Twenty-two participants (9%) reported a visit to an emergency room due to ingestion of aspirin or NSAID in the year prior to the survey.

**Table 1:** Demographic characteristic of survey respondents

Sex n (%)	Female	215 (87.76)
	Male	27 (11.02)
	Do not want to answer/no answer	3 (1.22)
Age—mean (SD)		51.13 (12.51)
Geographic Location	North America	191 (77.96)
	Europe	36 (14.69)
	Asia	4 (1.63)
	Africa	2 (0.82)
	Oceania	6 (2.45)
	No answer	6(2.45)
Highest Level of Education	Graduate High school	20 (8.16)
	Trade certificate/apprenticeship	6 (2.45)
	Community College/Other non-university program	55 (22.45)
	University Degree (Bachelor's)	85 (34.69)
	Postgraduate or Higher Degree	76 (31.02)
	No answer	3(1.22)
Household income	> $80,000 USD per year	125 (51.02)
	40,000–80,000 USD per year	60 (24.49)
	20,000–40,000 USD per year	23 (9.39)
	< $20, 000 USD per year	10 (4.08)
	No answer	27 (11.02)

**Table 2:** Types of Medical ID used by survey respondents

Type of ID	N (%) – multiple answers were possible
Bracelet	28 (59.57)
Smartphone technology	12 (25.53)
Wallet card	7 (14.89)
Watch	8 (17.02)
Necklace	6 (12.77)
Wristband	3 (6.38)
Tattoo	1 (2.13)

**Table 3:** Reason for not wearing an ID

Reason for not wearing an ID	n (%)
I did not know I should be wearing one	138 (69.70)
I don't think it is necessary	18 (9.09)
The cost of medical ID s too high	17 (8.59)
They are uncomfortable	13 (6.57)
I don’t want other people to know that I have a medical condition	8 (4.04)
Other (includes desensitization)	50 (25.25)

No associations were found between demographic characteristics and the use of medical ID (p > 0.05) or visits to the emergency room in the previous year due to accidental exposure to NSAIDs (> 0.05).

## Discussion

Accidental exposure to NSAIDs is not uncommon for patients with AERD, and in some instances it can lead to life-threatening reactions [4]. Patients diagnosed with AERD who are not desensitized must be educated on the names of the medications they must avoid. They should also be trained on the importance of relating this information to health care providers that may dispense or prescribe medications to them. It is also recommended that patients with AERD wear a medical ID. [1] This is a simple prevention strategy that can avoid accidental exposure to NSAIDs in an emergency situation. In our study, nearly 10% of the respondents had visited an emergency room within 12 months of the survey due to an adverse reaction to an NSAID. This suggests that annually, over 10% of patients with AERD may accidentally ingest an NSAID thereby putting them at risk for a severe medication adverse reaction.

Further, our study results demonstrated that 25% of respondents were aware that the use of medical ID is recommended for AERD patients, while only 19% respondents were using a medical ID at the time of the survey. The majority (81%) of respondents were not using any form of medical identification. Our current findings with this international cohort of AERD patients aligns with previous study results about medical ID use at our local institution, with an AERD population [8]. Despite differences in patient location, the attitudes towards medical ID use and the level of nonuse of medical ID are compellingly similar. The low prevalence of medical ID use could be explained by insufficient patient education by healthcare providers about the advantages of medical ID use for their NSAID sensitivity, representing a deficiency in patient care and a potential area for improvement. In this study, other reasons reported by survey respondents for not using a medical ID were high cost, not wanting others to know about their medical condition, appearance, and discomfort. Such reasons could be addressed and discussed with AERD patients with their health care provider during the education and counselling of the importance of using medical ID for their disease.

There was no association between age, income, geographical location, or education level and the use of medical ID in this study. However, there was an association between wearing medical ID and emergency room visits in the last year due to accidental exposure to NSAIDs. One potential explanation for this observation is the non-adherence phenomenon [10]. That is, the non-users of medical ID may have fewer health concerns and invest less in their in their own health. It is well known that nonadherence is a significant problem in chronic disease management [11] and non-adherence is directly related to elevated health care costs, hospitalizations, and patient mortality [12–14]. Despite this possible explanation, it remains a challenge to identify the exact cause for our results without having adequate information regarding the context of the visits to the emergency room or how the survey respondents were exposed to aspirin and NSAIDs.

The participants in this survey reported using many types of medical ID. The most commonly form of ID used was bracelets (60%) followed by smart phone technology (26%); other forms of ID such as a wallet card, wristbands and tattoos were also reported. The wide variety of options for medical ID can make it easier for patients to obtain one, but it can also mean that in an emergency, they are not easily located or accessible by health care providers. In our study, we did not ask for the type of information in the medical ID and therefore there may be limitations on the type and quality of the information provided. A recent study about the use of medical ID to communicate allergy information using of the MedicAlert Foundation database in Australia [15], found that the quality of the information on the ID was variable and non-standardized. The authors suggest that the specific allergen and nature of the reaction be recorded on the ID. Currently, there are no guidelines specifying what information AERD patients should have on a medical ID, therefore, this should be part of the education given to patients by their physicians.

There are clear advantages of using a medical ID for the AERD patient population to avoid or lower the risk of accidental exposure to aspirin and NSAIDs. Medical ID can serve as a reminder to AERD patients of their hypersensitivity when purchasing over counter medications or when getting a new physician prescription. The essential role of medical identification is clearly shown in emergencies when a patient cannot communicate to provide their medical history to health care providers, but they may [15] also expedite emergency treatment once an accidental exposure has occurred by providing information to emergency responders or emergency room personnel about possible cause for the hypersensitivity reaction. In our study, 9% of patients reported a visit to an emergency room due to exposure to aspirin or another NSAID, however the survey did not collect details about the circumstances around the accidental exposure and therefore we cannot determine if the use of medical ID could have prevented the exposure. The real impact of medical ID use by the AERD population remains unclear on their care. The present study demonstrated that AERD patients require more education regarding the importance of using medical ID, which in turn can be sign that health care providers need to be trained themselves on AERD, NSAID avoidance and the need for medical ID use in this population. Additionally, this study can be used as an impetus for more investment into patient safety initiatives and as a guide for additional work to prevent accidental exposure to aspirin and NSAIDs in the AERD patient population. Furthermore, future studies may be needed to evaluate both the real impact of medical ID in preventing accidental exposures or expediting treatment is needed as well as the cost-effectiveness of medical ID use by the AERD patient population.

Our study has some limitations. Although we asked those who answered the survey to confirm that they had been diagnosed with AERD, we could not verify their diagnosis. The Facebook support group has approximately 4500 members. We only obtained 245 complete responses to the survey (response rate of 5.4%). However, we do not know how many members of the group are actual patients with AERD, and how many may be family members or simply other interested members from the community. Given that AERD is more prevalent in females than males at a 3:1 ratio, we expected a similar distribution among survey respondents. However, the proportion of female respondents to our survey was higher than expected (88%). This may be explained by the fact that women have been found to use the internet more often than men to search health-related information and in particular, women use health forums and blogs more often than men [16]. Our survey was posted on the AERD Samter’s Triad Support Group on Facebook and it is possible that the majority of the members of the group are female.

## Conclusions

The findings of this study allow us to conclude that medical ID use in the AERD patient population is low. Further, patients lack the knowledge regarding the importance and advantages of using medical identification. The results of this study could be used for future research to improve patient safety and to evaluate the real impact and cost-effectiveness in the care of those with AERD.

## Supplementary Information

**Additional file 1. ** Survey

## Data Availability

The datasets used and analyzed during the current study are available from the corresponding author on reasonable request. The survey questions are available as Additional file 1.
