# Supplementary material for: Medical ID use by international patients with Aspirin-Exacerbated Respiratory Disease
Source: Allergy Asthma Clin Immunol. 2023 Mar 13;19:22. doi: 10.1186/s13223-023-00766-7 (PMC10012488; doi:10.1186/s13223-023-00766-7)
Supplement: Supplementary file 1 — Additional file 1. Survey [file 13223_2023_766_MOESM1_ESM.pdf]

LimeSurvey

Implied Consent Form

Study Title: Use of medical ID bracelet/necklace in patients with Aspirin-Exacerbated Respiratory Disease Principal Investigator: Dr. Shaun Kilty – 613-798-5555 ext. 18514 OHSN-REB Number: 20200267-01H <u>INTRODUCTION</u> You are being asked to participate because you have Aspirin Exacerbated Respiratory Disease (AERD). This study examines factors that prevent or motivate people with AERD to use or not use a medical ID/bracelet. <u>ARE THERE ANY CONFLICTS OF INTEREST?</u>

There are no conflicts of interest to declare related to this study.

<u>WHAT WILL HAPPEN DURING THIS STUDY?</u> Your participation in this study will require the completion of a survey. The survey asks questions about the reason why you wear/do not wear a medical ID. This should take approximately 10 minutes of your time. The information you provide is for research purposes only. Some of the questions are personal. You can choose not to answer questions if you wish.

<u>VOLUNTARY PARTICIPATION AND WITHDRAWAL:</u> You do not have to be in this study if you do not want to be. You can choose to end your participation in this research (called withdrawal) at any time without having to provide a reason.

The survey is anonymous. This means that you can withdraw from participating at any time while completing the survey simply by closing your browser; however, once the completed survey has been returned to the study team, it will not be possible to withdraw your information and the information recorded in the completed survey will be used by the researchers for the purposes of the study.

<u>RISKS AND/OR BENEFITS</u> Participation involves minimal risk to you. Some of the questions may however make you feel uncomfortable. You may not receive direct benefit from participating in this study. We hope the information learned from this study will help other people with AERD in the future.

<u>PRIVACY/CONFIDENTIATLITY:</u>

The survey is anonymous which means that your answers will not be linked to you in any way.

Authorized representatives of the following organizations may look at your original research records at the site where these records are held, to check that the information collected for the study is correct and follows proper laws and guidelines.

The Ottawa Health Science Network Research Ethics Board who oversees the ethical

LimeSurvey

Section A: Questions

A1. **Thank you for agreeing to participate in this study. The objective of this study to explore the opinions and attitudes of people who have Aspirin Exacerbated Respiratory Disease about the use of Medical Alert ID. A medical alert ID is an identifier that a person wears or has with them which bears a message to alert medical personnel that the wearer has an important medical condition in the event the wearer is unable to communicate that condition themselves.**

This survey should take about 5 - 10 minutes to complete. You don't have to answer any questions that make you uncomfortable.

Please confirm that you have been diagnosed with Aspirin Exacerbated Respiratory Disease:

Yes ☐

No ☐

A2. **Sex:**

Female ☐

Male ☐

Other ☐

Do not want to answer ☐

A3. **Month of Birth**

- January ☐
- February ☐
- March ☐
- April ☐
- May ☐
- June ☐
- July ☐
- August ☐
- September ☐
- October ☐
- November ☐
- December ☐

A4. **Year of birth**

☐☐☐☐☐☐☐☐☐☐

A5. **Please mark the region where you are currently located: (optional)**

- Africa ☐
- North America ☐
- Latin America and the Caribbean ☐
- Asia ☐
- Europe ☐
- Oceania ☐

A6. **What is the highest level of education that you have completed? (optional)**

- Grade 8 or less ☐
- Some high school but did not graduate ☐
- Graduated high school ☐
- Trade certificate/apprenticeship ☐
- Community College/other non-university program ☐
- University Degree (Bachelor's) ☐
- Postgraduate or Higher Degree ☐

A7. **Which income bracket best describes your household family income (or, if you are single, your income). (optional)**

- < $20, 000 USD per year ☐
- 20,000-40,000 USD per year ☐
- 40,000-80,000 USD per year ☐
- > $80,000 USD per year ☐

A8. **Are you aware that for people with Aspirin Exacerbated Respiratory Disease (AERD) it is recommended to wear a Medical Alert ID?**

- Yes ☐
- No ☐

A9. **Do you currently wear a Medical Alert ID or use any kind of medical alert?**

- Yes ☐
- No ☐

A10. **What kind of Medical Alert ID do you use? (check all that apply)**

- Bracelet ☐

Comment

- Necklace ☐

Comment

LimeSurvey

Ankle tag

Comment

Watch

Comment

Wristband

Comment

Other Type of jewelry (please specify)

Comment

Tattoo

Comment

Smart Phone technology

Comment

Other type of technology (please specify)

Comment

Wallet Card

Comment

LimeSurvey

Other system, (please specify any other medical alert ID used)

Comment

A11. **How long have you been wearing the alert for?**

- < 1 year
- 1-5 years
- 5-10 years
- > 10 years

A12. **Are there any particular reasons why you don't wear Medical Alert ID? (check all that apply)**

- I did not know I should be wearing one.
- The cost of medical ID is too high.
- I don't want other people to know that I have a medical condition.
- I don't think it is necessary.
- They are uncomfortable.
- Other

Other

A13. **In the past year, have you required an emergency room visit due to exposure to ASA/NSAIDs?**

- Yes
- No

Section B: Information sheet

Information sheet

LimeSurvey

This is the end of the survey. Thank you for participating.
